# Access to Care and Outcomes With the Affordable Care Act for Persons With Criminal Legal Involvement: A Scoping Review

**DOI:** 10.1001/jamahealthforum.2024.2640

**Published:** 2024-08-23

**Authors:** James René Jolin, Benjamin A. Barsky, Carrie G. Wade, Meredith B. Rosenthal

**Affiliations:** 1 Harvard College, Cambridge, Massachusetts; 2 University of California College of the Law, San Francisco; 3 Interfaculty Initiative in Health Policy, Harvard University, Cambridge, Massachusetts; 4 Countway Library, Harvard Medical School, Boston, Massachusetts; 5 Department of Health Policy and Management, Harvard T.H. Chan School of Public Health, Boston, Massachusetts

## Abstract

**Importance:**

By expanding health insurance to millions of people in the US, the Patient Protection and Affordable Care Act (ACA) may have important health, economic, and social welfare implications for people with criminal legal involvement—a population with disproportionately high morbidity and mortality rates.

**Objective:**

To scope the literature for studies assessing the association of any provision of the ACA with 5 types of outcomes, including insurance coverage rates, access to care, health outcomes, costs of care, and social welfare outcomes among people with criminal legal involvement.

**Evidence Review:**

The literature search included results from PubMed, CINAHL Complete, APA Psycinfo, Embase, Social Science Database, and Web of Science and was conducted to include articles from January 1, 2014, through December 31, 2023. Only original empirical studies were included, but there were no restrictions on study design.

**Findings:**

Of the 3538 studies initially identified for potential inclusion, the final sample included 19 studies. These 19 studies differed substantially in their definition of criminal legal involvement and units of analysis. The studies also varied with respect to study design, but difference-in-differences methods were used in 10 of the included studies. With respect to outcomes, 100 unique outcomes were identified across the 19 studies, with at least 1 in all 5 outcome categories determined prior to the literature search. Health insurance coverage and access to care were the most frequently studied outcomes. Results for the other 3 outcome categories were mixed, potentially due to heterogeneous definitions of populations, interventions, and outcomes and to limitations in the availability of individual-level datasets that link incarceration data with health-related data.

**Conclusions and Relevance:**

In this scoping review, the ACA was associated with an increase in insurance coverage and a decrease in recidivism rates among people with criminal legal involvement. Future research and data collection are needed to understand more fully health and nonhealth outcomes among people with criminal legal involvement related to the ACA and other health insurance policies—as well as the mechanisms underlying these relationships.

## Key Points

**Question:**

How did insurance coverage, access to health care, health outcomes, costs of care, and social welfare outcomes change among people with criminal legal involvement after the Patient Protection and Affordable Care Act (ACA)?

**Findings:**

In this scoping review, the ACA was associated with an increase in insurance coverage and a decrease in recidivism among people with criminal legal involvement. For other outcomes, evidence was scant or mixed.

**Meaning:**

The ACA was associated with improved insurance coverage and reduced recidivism outcomes among people with criminal legal involvement, and further research is needed to assess health care use, health outcomes, and costs of care.

## Teaser

This scoping review assesses the association of any provision of the Patient Protection and Affordable Care Act with 5 types of outcomes, including insurance coverage rates, access to care, health outcomes, costs of care, and social welfare outcomes, among people with criminal legal involvement.

## Introduction

Enacted in 2010, the Patient Protection and Affordable Care Act (ACA) expanded health insurance to millions of people in the US.^1,2^ Among other provisions, the ACA enabled state Medicaid programs to cover all low-income adults up to 138% of the federal poverty level.^1,3,4^ Its dependent coverage mandate required private health insurance plans to allow adult children aged 19 to 25 years to stay on their parents’ plans.^1^ It also introduced health insurance marketplaces, which expanded coverage to individuals without other health insurance.^1^ Studies have found the ACA was associated with increased insurance coverage rates, improved health outcomes, increased political participation, and decreased medical debt for the general population.^5,6,7,8^

However, less is known about the outcomes of persons with criminal legal involvement after the ACA coverage expansions were implemented. People with criminal legal involvement stood to benefit from the ACA because this population is disproportionately composed of individuals with low incomes who have been historically uninsured or underinsured.^9,10,11^ Accordingly, this review scopes the literature for studies of insurance coverage, access to or utilization of health care, costs of care, and social welfare outcomes among people with criminal legal involvement related to the ACA. Furthermore, given that this population is disproportionately affected by chronic disease,^2,12^ we also explore health outcomes for this population.

## Methods

We conducted a scoping review in lieu of a systematic review because no other published literature reviews, to our knowledge, have assessed outcomes of the ACA among people with criminal legal involvement. Scoping reviews have become increasingly common in the public health and medical literature and aim to map “key concepts, types of evidence, and gaps in research related to a defined area or field by systematically searching, selecting, and synthesizing existing knowledge.”^13^ This scoping review was conducted according to the JBI Manual for Evidence.^14^ This scoping review was exempted from institutional review board review because it does not constitute human subjects research. This study followed the Preferred Reporting Items for Systematic Reviews and Meta-analyses (PRISMA) reporting guideline.

Because of the exploratory nature of this review, we broadly defined the intervention, population, and outcomes of interest. Doing so allowed us to identify and investigate as many studies as possible. Specifically, we defined the intervention as any provision of the ACA. We defined the population of interest as any individuals of any age who are or have been involved in the criminal legal system, including those who have been or are incarcerated in prisons, jails, and other detention facilities; those who have been or are on parole or probation; and those who have been arrested, charged, or convicted. We defined primary outcomes as including insurance coverage rates, access to care, mental and physical health outcomes, cost of care, and social welfare outcomes among people with criminal legal involvement. We describe these categories, provide examples, present actual definitions from included studies in (eTable 1 in Supplement 1). As we scoped the literature, we adjusted the definition of our primary outcomes to allow flexibility in the review process.

### Study Identification

In consultation with an experienced medical librarian (C.G.W.), we developed a search strategy iteratively and deposited it into an Open Science Framework project (eTables 2 and 3 in Supplement 1). We included results from searching PubMed, CINAHL Complete, APA Psycinfo, Embase, Social Science Database, and Web of Science on December 31, 2023.

We included any study that assessed outcomes of the ACA related to health insurance coverage rates, access to care, cost of care, health outcomes, and social welfare outcomes among people with criminal legal involvement. We excluded nonempirical articles. Review articles, conference proceedings, studies with unclear methodologies, any non–peer-reviewed reports, and articles inaccessible through our institutional libraries were also excluded. We limited results to those written in English and published between January 1, 2014, through December 31, 2023. Results were exported to EndNote, then deduplicated. The Figure summarizes our search and screening process.

**Figure.**
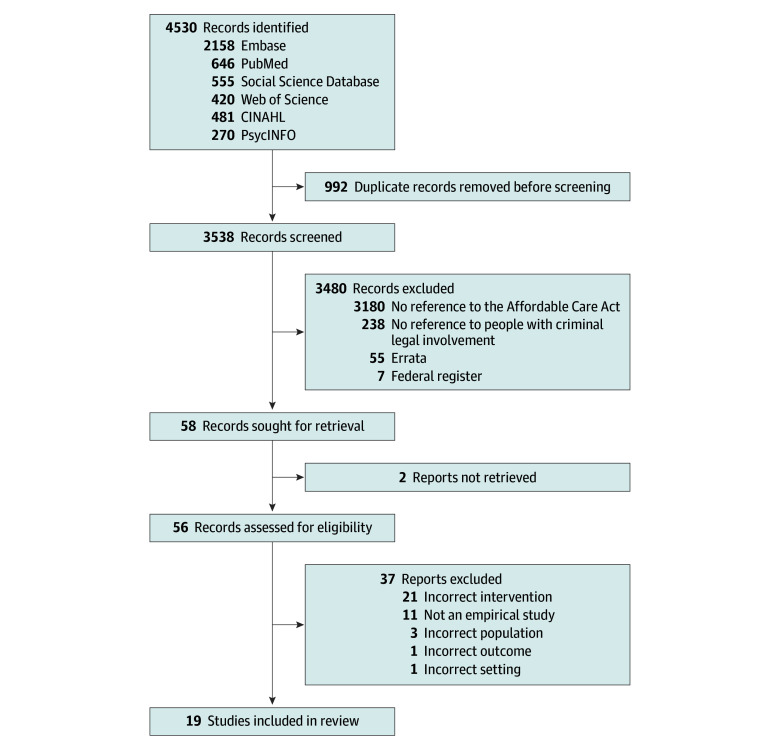
PRISMA Flowchart

### Study Selection

Two of us (J.R.J. and B.A.B.) independently screened identified studies to exclude those that did not meet inclusion criteria using Covidence 2023 literature review software (Covidence). In consultation with B.A.B., 1 reviewer (J.R.J.) subsequently examined the full-text articles to identify those that assess the association or causal effect of ACA on outcomes of interest. Reviewer conflicts were resolved through reviewer discussion or with the aid of a third reviewer (M.B.R.) on an ad hoc basis.

### Data Extraction and Synthesis

Data from studies identified as eligible for inclusion were extracted using templates within Covidence. After securing the full text of each included study, 1 author (J.R.J.) extracted the following data: authors of study, year of publication, journal, study design, unit(s) of analysis, number of control or treated units, sample size, population, years of outcome analyzed, type of outcome assessed, data sources, key findings and conclusions from analysis, and study limitations. These data were compiled in a data extraction table within Covidence.

One reviewer (J.R.J.) performed initial data extraction. Included studies underwent meta-aggregative qualitative data synthesis as outlined in the JBI Manual for Evidence.^14^ One reviewer (J.R.J.) developed categories for sufficiently similar findings and synthesized findings. Two reviewers (B.A.B. and M.B.R.) independently reviewed the results of data extraction and synthesis once complete.

## Results

### Study Characteristics

eTable 4 in Supplement 1 presents the study characteristics across all 19 included studies. Seven studies (37%) reported using the National Survey on Drug Use and Health (NSDUH) as a primary data source,^15,16,17,18,19,20,21^ while 3 studies (16%) reported using the Treatment Episode Data Set–Admissions (TEDS-A) dataset for their analyses.^22,23,24^ Only 1 study fielded its own survey to collect data.^25^ Using the outcome with the longest study period for each included study, the mean study period was approximately 8.5 years, with a range of study years of 1 to 26 years.

Studies differed substantially in their definition of criminal legal involvement, their units of analysis (eg, person-level vs county-level), and the subpopulations included. For example, while 1 study analyzed only male prisoners aged 18 to 64 years,^26^ another study analyzed only pregnant women with opioid use disorder (OUD) referred to OUD treatment by criminal justice agencies.^22^ Due to different sample sizes, the statistical power of included studies also varied widely. Studies also varied in design, although difference-in-differences methods were used in 10 of the included studies.^15,19,20,22,24,27,28,29,30,31^ With respect to outcomes, we identified 100 unique outcomes in 5 categories (eTable 4 in Supplement 1). Across these 100 outcomes, 31 (31%) outcomes were related to access to care, 38 (38%) were related to insurance coverage, 21 (21%) were related to social welfare, 6 (6%) were related to health outcomes, and 4 (4%) were related to costs of care.

### Insurance Coverage

Of the 19 included studies, 11 (58%) assessed how the ACA was associated with changes with insurance coverage.^15,16,17,18,19,20,21,25,26,32,33^ Winkelman et al^15^ found that the ACA was associated with lower uninsurance rates and increased private insurance, marketplace plans, and Medicaid enrollment rates among people with criminal legal involvement. In a separate study, Winkelman and colleagues^32^ also showed that the ACA was associated with significantly reduced uninsurance rates and increased private insurance rates among people with criminal legal involvement. Saloner and colleagues^16^ also found that the ACA was associated with lower uninsurance rates and higher enrollment rates in Medicaid and Medicare among people with criminal legal involvement.

Similarly, Knapp et al^17^ demonstrated that the ACA was associated with higher insurance coverage rates and Medicaid insurance coverage rates among people with criminal legal involvement. These results aligned with a study by Howell et al^18^ that showed that the ACA was associated with a higher proportion of people with criminal legal involvement with private insurance. In a later study, Howell et al^19^ showed that Medicaid expansion was associated with increased insurance coverage rates in general and Medicaid coverage rates in particular. In yet another study, Howell et al^21^ found that the ACA corresponded to a significant increase in insurance coverage rates among NSDUH survey respondents with household incomes of 138% or less of the federal poverty level and with either a substance use disorder (SUD) or mental health disorder. Also leveraging NSDUH data, Gutierrez and Pettit^20^ found that the ACA was associated with lower uninsurance rates. Using a panel survey of minors and young adults, Testa and Porter^33^ yielded similar results, finding that Medicaid expansion was associated with a higher likelihood of being insured under a public program.^33^ These increases appeared to be largest among White respondents. Finally, among a sample of formerly incarcerated women in Kentucky, Dickson and colleagues^25^ found that women released after ACA implementation (January 2014 to August 2015) were more likely to be insured relative to those released prior to implementation (November 2012 to December 2013).

In contrast to other studies, Rosen and colleagues^26^ projected how insurance coverage eligibility among people released from prison might change after the ACA, using the 2004 Survey of Inmates in State Correctional Facilities. They estimated that 20% and 17% of those in Medicaid expansion and nonexpansion states, respectively, would earn greater than or equal to 400% of the federal poverty level and thus would be ineligible for Medicaid coverage or ACA marketplace tax credits.^26^ In expansion states, they reported 73% would qualify for Medicaid or tax credits, while in nonexpansion states, only 60% would be eligible for ACA-related benefits. Moreover 22% of people released from prison in nonexpansion states would fall into the coverage gap (ie, failing to qualify for tax credits by virtue of having incomes at or equal to 100% of the federal poverty level).^26^

### Access to Care

A total of 11 studies (58%) assessed how the ACA was associated with outcomes related to access to care.^15,16,18,20,21,22,23,24,25,30,32^ Some studies reported access to care improved after ACA, but results were not consistent. Winkelman and colleagues^22^ found Medicaid expansion was associated with higher likelihood of receiving medications for OUD among a subset of pregnant women with OUD referred to treatment by criminal justice agencies. These results concurred with Sledge et al^23^ who found the odds of SUD treatment for admissions in Medicaid expansion states were significantly higher than in nonexpansion states but with significant racial heterogeneity. Similarly, Khatri and colleagues^24^ reported that Medicaid expansion was associated with an increase in the receipt of medications for OUD between 2008 and 2017 for individuals referred to criminal justice agencies. Finally, Aslim et al^30^ found that Medicaid expansion was associated with an increase in the frequency of admissions to SUD treatment.

Winkelman et al^15^ found the increased private and Medicaid insurance coverage rates among people with criminal legal involvement after the ACA were associated with SUD and mental illness treatment. However, Saloner et al^16^ reported that, after ACA, rates of SUD treatment among people with criminal legal involvement in any setting did not change significantly. Likewise, after ACA implementation, Howell et al^18^ found no significant change in receipt of any mental illness treatment among people with criminal legal involvement. Furthermore, in a later study, Howell et al^21^ found receipt of inpatient or outpatient treatment for drug or alcohol use was statistically unchanged after Medicaid expansion among those with SUD in their sample.

Gutierrez and Pettit^20^ used a quasi-experimental difference-in-differences approach and found a significant reduction in the percentage of people with criminal legal involvement reporting a hospital stay, while the percentage reporting an emergency department visit was unchanged. Similarly, Dickson and colleagues^25^ observed that a significantly larger proportion of their study participants reported having a usual source of nonemergency, outpatient health care after ACA. They also found the proportion of participants reporting having gone to the emergency department for health problems during the 3-month follow-up period was significantly reduced after ACA.^25^

### Health Outcomes

Only 3 studies (16%) assessed health outcomes among people with criminal legal involvement.^18,20,25^ Howell et al^18^ reported a statistically insignificant decrease in the proportion of individuals with unmet mental health care needs following ACA implementation from the 2011 to 2013 period to the 2014 to 2017 period. Likewise, Dickson et al^25^ found a statistically insignificant difference in the number of days individuals reported being bothered by health problems during the 3-month follow-up period between those released before and after ACA implementation. Gutierrez and Pettit,^20^ by contrast, assessed the rate of diagnosis or reporting of particular diseases among recently incarcerated male respondents to the NSDUH aged 18 to 64 years. They found a significant decline in diagnosed hypertension, but they did not find significant changes in diagnosed diabetes, reported mental illness, or reported SUD.^20^

### Costs of Care

Only 2 studies (11%) assessed the association between the ACA and cost of care among people with criminal legal involvement.^16,18^ Howell et al^18^ did not find significant changes in the proportion of people with criminal legal involvement reporting that they did not receive care because of financial reasons, that Medicaid paid for their mental health treatment, or that their mental health treatment was paid for by themselves or their family members. By contrast, Saloner et al^16^ found the ACA was associated with a statistically significant increase in the proportion of people with criminal legal involvement reporting Medicaid paid for their costs of care.

### Social Welfare

With respect to social welfare outcomes, 5 quasi-experimental investigations studied changes in recidivism rates and crime rates after ACA Medicaid expansion.^27,28,29,30,31^ Unlike other included studies, many of these studies focused on counties or states and not individual survey respondents.

Simes and Jahn^27^ used a county-level difference-in-differences design, facilitated by differences in uptake of Medicaid expansion across US states, to investigate the rate of arrest, arrests for violent offenses, low-level arrests, and drug-related arrests between counties in Medicaid expansion states and nonexpansion states from 2011 to 2016. They found that Medicaid expansion was associated with reductions in all of these crime-related outcomes.^27^ Likewise, He and Barkowski^28^ examined property, burglary, larceny, motor vehicle, violent crime, criminal homicide, robbery, and aggravated assault crime rates between Medicaid expansion and nonexpansion US states (including Washington, DC) and a sample of counties. Medicaid expansion was associated with a decrease in 5 of these outcomes (burglary, motor vehicle, violent crime, criminal homicide, and robbery).^28^

In a case study, Fry et al^29^ examined 48 continuous months of individual-level booking and release dates from 6 urban county jails to estimate an average change in probability of rearrest in pairs of Midwest states, Southwest states, and Southeast treatment control county pairs. Their analyses yielded mixed results, finding Medicaid expansion was associated with a decrease in average rearrest rates in only the Midwest and Southwest case studies.^29^ Finally, Aslim and colleagues^30^ used a difference-in-differences design and found the rates of individual returns to prison within 1 and 2 years of release were both significantly lower in Medicaid expansion vs nonexpansion states.

One quasi-experimental study assessed how the ACA was associated with crime rates. Fone et al^31^ found that the ACA’s dependent coverage mandate was associated with lower rates of property crime arrests involving individuals aged 22 to 25 years.

## Discussion

We identified 19 observational and quasi-experimental studies assessing outcomes relating to health insurance coverage, access to care, health outcomes, costs of care, and social welfare outcomes among people with criminal legal involvement after the ACA. Results were mixed, potentially due to heterogeneous definitions of populations, interventions, and outcomes and to limitations in the availability of individual-level datasets linking incarceration data with health-related data.

Many studies found that implementation of the ACA was associated with lower uninsurance rates among people with criminal legal involvement, but whether private or public insurance rates increased correspondingly was less clear. With respect to access to care, many studies focused on substance use and mental illness treatment among people with criminal legal involvement but diverged in whether the ACA was associated with improved access to these forms of care. The quasi-experimental studies assessing Medicaid expansion’s association with recidivism and crime rates were more convergent, suggesting both outcomes decreased after ACA. By contrast, studies provide only suggestive evidence that some health and cost of care outcomes among people with criminal legal involvement changed after ACA. Despite the absence of consistent health benefits for people with criminal legal involvement in a limited number of studies since implementation of the ACA coverage expansions, recidivism appeared to improve, suggesting that non–health-related mechanisms (eg, financial security associated with insurance) may explain these results.

Broadly, our findings suggest that interventions expanding access to health insurance coverage to low-income people can improve outcomes among people with criminal legal involvement. Recently, the US Centers for Medicare and Medicaid Services approved Section 1115 requests by California, Washington, and Montana to waive partially the longstanding Medicaid Inmate Exclusion Policy and cover certain reentry services for incarcerated people prior to release.^34,35,36,37^ Our review suggests these waivers may yield positive effects, especially if they facilitate Medicaid enrollment on release and connection with community-based health care. However, our results emphasize that improved measurement of health and social outcomes among people with criminal legal involvement is critical to evaluate these interventions.

### Limitations

This article has several limitations. First, our review may not include all analyses of the association between the ACA and health among people with criminal legal involvement. Second, because of the exploratory nature of our study, we defined the intervention, population, and outcomes of interest broadly to capture as many studies as possible. Variation in these definitions limits cross-study comparability and constrains our ability to draw conclusive implications for research and policy. Third, our review does not explicitly address quality of care, only access to care. Investigating how quality of care for people with criminal legal involvement changed following the ACA may qualify or help clarify the conflicting results we find with respect to access to care. Fourth, more than half of our included studies draw from only 2 data sources: the NSDUH and TEDS-A datasets. This fact is likely because the linkage of health and criminal legal data are rare, and NSDUH is one of the only surveys to collect both data types simultaneously. Finally, our study did not examine the particular mechanisms linking the ACA and its constituent provisions to the outcomes analyzed within our population of interest.

## Conclusions

In this scoping review, we summarized 19 observational and quasi-experimental studies assessing the association between the ACA and outcomes relating to access to care, health insurance, health, social welfare, and costs of care among people with criminal legal involvement. Results were mixed, potentially due to heterogeneous definitions of population, interventions, and outcomes and to limitations in the availability of incarceration data linked with health-related data. However, many studies show that the ACA was associated with higher insurance coverage and reduced recidivism among people with criminal legal involvement.

Future research should assess the health and social effects of the ACA among this population, particularly related to health outcomes and costs of care, for which evidence is scant. Researchers can also probe the mechanisms of the ACA’s association with improved insurance coverage and reduced recidivism among people with criminal legal involvement. To this end, researchers may find prudent to leverage the variation in prerelease Medicaid enrollment assistance under some states’ Social Security Act Section 1115 demonstrations, which directly connect people with criminal legal involvement to Medicaid coverage.^38,39,40^ Such Medicaid enrollment assistance programs have the potential to increase enrollment rates and use of health care. To do so, however, researchers will need additional datasets that contain both health and criminal-legal history data.

## Notes

Supplement 1. **eTable 1.** Outcome Categories, Examples, and Included Outcomes **eTable 2.** Concepts and Keywords **eTable 3.** Search Strategy and Results, Conducted December 31, 2023 **eTable 4.** Description of Included Studies, Including Provision of Patient Protection and Affordable Care Act (ACA) Analyzed, Data Source, Study Period, Sample, Design, and Outcomes
eReferences.

Supplement 2. Data Sharing Statement
