# Supplementary material for: Access to Care and Outcomes With the Affordable Care Act for Persons With Criminal Legal Involvement: A Scoping Review
Source: JAMA Health Forum. 2024 Aug 23;5(8):e242640. doi: 10.1001/jamahealthforum.2024.2640 (PMC11344231; doi:10.1001/jamahealthforum.2024.2640)
Supplement: Supplement 1. — eTable 1. Outcome Categories, Examples, and Included Outcomes eTable 2. Concepts and Keywords eTable 3. Search Strategy and Results, Conducted December 31, 2023 eTable 4. Description of Included Studies, Including Provision of Patient Protection and Affordable Care Act (ACA) Analyzed, Data Source, Study Period, Sample, Design, and Outcomes eReferences. [file jamahealthforum-e242640-s001.pdf]

Supplemental Online Content

Jolin JR, Barsky BA, Wade CG, Rosenthal MB. Access to care and outcomes with the Affordable Care Act for persons with criminal legal involvement: a scoping review. *JAMA Health Forum.* 2024;5(8):e242640. doi:10.1001/jamahealthforum.2024.2640

eTable 1. Outcome Categories, Examples, and Included Outcomes
eTable 2. Concepts and Keywords
eTable 3. Search Strategy and Results, Conducted December 31, 2023
eTable 4. Description of Included Studies, Including Provision of Patient Protection and Affordable Care Act (ACA) Analyzed, Data Source, Study Period, Sample, Design, and Outcomes
eReferences.

This supplementary material has been provided by the authors to give readers additional information about their work.

© 2024 Jolin JR et al. *JAMA Health Forum*.

eTable 1. Outcome Categories, Examples, and Included Outcomes

Outcome Category	Example Outcomes Conceptualized Prior to Analysis	Actual Outcomes from Included Studies
Access to care	Timeliness of care among PCLI (e.g., appointment availability or wait time), use of health care services among PCLI, hospital readmissions among PCLI, or emergency room visits among PCLI	• The extent to which medications for OUD changed among pregnant women with a criminal justice referral with OUD in states that expanded Medicaid.<br>• The extent to which pregnant women referred by criminal justice agencies were more or less likely to have received medications for OUD in states that expanded Medicaid compared with women in states that did not expand Medicaid<br>• Medicaid and private insurance association with rates of treatment among justice-involved individuals with serious mental illness compared to those without insurance<br>• Among justice-involved individuals with alcohol use/dependence, Medicaid and private insurance association with rates of treatment<br>• Medicaid effect on SUD treatment among those with illicit drug use/dependence<br>• Difference in reporting regular source of primary care after ACA implementation, relative to men without history of incarceration<br>• Reporting using ED as regular source of care or for any reason after ACA expansion, compared to never incarcerated<br>• Odds of treatment for admissions in states with Medicaid expansion<br>• Odds of MAT in Medicaid expansion states for criminal justice-referred admission<br>• Odds of treatment for admissions in states with Medicaid expansion, for Native American, Asian, Black, Hispanic, Other relative to non-Hispanic white<br>• Odds of MAT in Medicaid expansion states for criminal justice-referred admission for Black relative to White<br>• Odds of MAT in Medicaid expansion states for criminal justice-referred admission for Hispanic relative to White<br>• Share of individuals with substance use disorder and criminal justice involvement receiving substance use disorder treatment (in any setting)<br>• Change in treatment setting among those with substance use disorder who received treatment<br>• Among individuals referred by criminal justice agencies, rates of medications for OUD receipt among those in Medicaid expansion states relative those in non-expansion states<br>• Between 2008-13 and 2015-17, change in receipt of medications for OUD among individuals referred by the criminal justice agencies in states that expanded Medicaid compared with such individuals in states that did not expand Medicaid<br>• Adjusted rates of medications for opioid use disorder for individuals in the US referred to treatment in Medicaid expansion and non-expansion states, by treatment referral source, 2008–17, among non-Hispanic White, non-Hispanic Black, Hispanic, Native American/Alaska Native, other race populations<br>• Adjusted rates of medications for opioid use disorder for individuals in the US referred to treatment in Medicaid expansion and nonexpansion states, by treatment referral source, 2008–17, in ambulatory, residential settings<br>• Among adults with past-year criminal justice involvement, change in receipt of any mental health treatment following ACA implementation<br>• Among individuals with criminal justice involvement in the past year, change in the proportion reporting any inpatient mental health treatment, outpatient mental health treatment, receipt of prescription medication for a mental disorder<br>• Hospital stay past 12 months<br>• ED visit past 12 months<br>• % have usual source of nonemergency, outpatient health care<br>• % currently being treated for a health problems<br>• % went to ER for health problems during the 3-month follow-up period<br>• % went to substance use treatment program during the 3-month follow-up period

© 2024 Jolin JR et al. *JAMA Health Forum.*

		• Availability of services (M; 1=not at all available, 10=extremely available)<br>• Having a usual source of non-emergency, outpatient care at 3 month follow- up<br>• Count of admissions to SUD treatment, aggregated on the state level<br>• Proportion of sample with substance use disorders reporting receipt of inpatient or outpatient treatment for illicit drug or alcohol use in the past year<br>• Proportion of sample with other mental health disorders reporting receipt of inpatient, outpatient, or pharmacy services for mental health in the past year
Insurance coverage	The proportion of PCLI covered by some form of health care insurance	• Effect of the dependent coverage provision on uninsurance rates<br>• Effect of the dependent coverage provision on private insurance rates<br>• Effect of the dependent coverage provision on Medicaid coverage rates<br>• Effect of Medicaid expansion and Marketplace plans in 2014 on uninsurance rates<br>• Effect of Medicaid expansion and Marketplace plans in 2014 on Medicaid enrollment<br>• ACA effect on uninsurance rate<br>• ACA effect on private insurance rate<br>• ACA effect on public insurance rate<br>• ACA effect on gaps in insurance coverage<br>• Uninsurance rate<br>• Medicaid enrollment<br>• Medicare enrollment<br>• Projected proportion of prisoners earning greater than or equal to 400% FPL and thus would be ineligible for Medicaid or tax credits<br>• In expansion states, projected proportion of prisoners qualifying for Medicaid or tax credits<br>• In non-expansion states, proportion of prisoners qualified for tax credits, but were projected to have incomes 100% FPL and would not qualify for any support (Fig 1), placing them in the coverage gap<br>• In non-expansion states, proportion of prisoners qualified for tax credits<br>• Change in insurance coverage rates between 2013 and 2016<br>• Changes in private health insurance, other insurance coverage rates between 2013 and 2017<br>• Changes in Medicaid insurance coverage rates between 2013 and 2017<br>• Proportion of individuals who had insurance coverage among those who reported criminal justice involvement in the past year<br>• Proportion of individuals with criminal justice involvement in the past year who had Medicaid insurance<br>• Proportion of individuals with criminal justice involvement who had private insurance<br>• Insurance coverage<br>• Medicaid coverage<br>• Private insurance coverage<br>• Other forms of coverage, Medicare, Tricare, or VHA<br>• Health uninsurance rate<br>• % insured for at least one month during 3-month follow-up period<br>• Proportion of sample with substance use disorders reporting being enrolled in a private or public health insurance plan<br>• Proportion of sample with other mental health disorder reporting being enrolled in a private or public health insurance plan<br>• Odds of being insured, for those with a history of being incarcerated<br>• Odds of being insured under public insurance program, for those with a history of being incarcerated<br>• Odds of being insured, for those with a history of being incarcerated, among White respondents<br>• Odds of being insured, for those with a history of being incarcerated, among Black respondents

© 2024 Jolin JR et al. *JAMA Health Forum*.

		• Odds of being insured, for those with a history of being incarcerated, among Hispanic respondents<br>• Odds of being insured under public insurance program, for those with a history of being incarcerated, among White respondents<br>• Odds of being insured under public insurance program, for those with a history of being incarcerated, among Black respondents<br>• Odds of being insured under public insurance program, for those with a history of being incarcerated, among Hispanic respondents
Health outcomes	Morbidity and mortality from chronic diseases (e.g., heart disease, stroke, diabetes, cancer) among PCLI, morbidity and mortality from substance-use disorders (e.g., alcohol-use disorder, opioid-use disorder) among PCLI, self-reported health quality among PCLI, morbidity and mortality from mental health disorders (including schizophrenia, depression, bipolar disorder)	• Change in the proportion of individuals with unmet mental health care needs following ACA implementation among individuals with criminal justice involvement in the past year<br>• Diabetes<br>• Hypertension<br>• Mental illness<br>• Substance use disorder<br>• No. of days bothered by health problems during 3-month follow-up period
Social welfare outcomes	Total financial debt held by PCLI, credit scores among PCLI, unemployment rates among PCLI, worker productivity among PCLI, self-reported quality of life among PCLI, voter turnout rates among PCLI, self-reported political efficacy among PCLI, or recidivism among PCLI	• Rate of arrest in counties in expansion states compared to counties in non-expansion states<br>• Difference in arrests for violent arrests in all three years of Medicaid expansion<br>• Difference in low-level arrests between expansion states compared to non-expansion state<br>• County-level drug-related arrests in expansion counties relative to counties in non-expansion states<br>• Natural log of property crime rate per 100,000 residents<br>• Natural log of burglary crime rate per 100,000 residents<br>• Natural log of larceny theft crime rate per 100,000 residents<br>• Natural log of motor vehicle crime rate per 100,000 residents<br>• Natural log of violent crime rate per 100,000 residents<br>• Natural log of criminal homicide crime rate per 100,000 residents<br>• Natural log of robbery crime rate per 100,000 residents<br>• Natural log of aggravated assault crime rate per 100,000 residents<br>• Average change in probability of rearrest in Midwest treatment-control county pair<br>• Average change in probability of rearrest in Southwest treatment-control county pair<br>• Average change in probability of rearrest in Southeast treatment-control county pair<br>• Whether inmate returns to prison within one year of release<br>• Whether inmate returns to prison within two years of release

© 2024 Jolin JR et al. *JAMA Health Forum*.

		• Number of property crime arrests involving 22–25-year-olds during the enactment period<br>• Number of property crime arrests involving 22–25-year-olds in the postimplementation period<br>• Number of violent crime arrests involving 22–25-year-olds during the enactment period<br>• Number of violent crime arrests involving 22–25-year-olds in the postimplementation period
Costs of care	Total health care spending among PCLI per year, average health care spending among PCLI per month, out-of-pocket costs among PCLI per year or disparities in health care costs among PCLI	• Source of payment for treatment being Medicaid<br>• Among those PCJI who reported an unmet mental health need, change in those reporting that they did not get care because of financial reasons<br>• Among PCJI, change in those reporting that Medicaid paid for their mental health treatment<br>• Among PCJI, change in the proportion that reported their mental health treatment was paid for by themselves or their family members

© 2024 Jolin JR et al. *JAMA Health Forum*.

eTable 2. Concepts and Keywords

Concept	Keywords	MeSH Terms	PubMed Search Example
Healthcare Reform	Affordable Care act<br>PL 111-148<br>PL111-148"<br>ACA<br>"healthcare reform"<br>"health care reform"<br>Obamacare<br>"obama care"<br>"patient protection act*"<br>"section 1115"<br>"dependent coverage"<br>medicaid	"Patient Protection and Affordable Care Act"<br>"Medicaid"	("Patient Protection and Affordable Care Act"[Mesh] OR "Medicaid"[Mesh]) OR ("111-148"[Title/Abstract] OR "PL111-148"[Title/Abstract] OR "affordable care act*"[Title/Abstract] OR ACA[Title/Abstract] OR "health care reform*"[Title/Abstract] OR "healthcare reform*"[Title/Asbract] OR obamacare[Title/Abstract] OR "obama care"[Title/Abstract] OR "patient protection act*"[Title/Abstract] or "section 1115" [Title/Abstract] OR "dependent coverage"[Title/Abstract] OR "medicaid"[Title/Abstract])
Justice-Involved Persons	Carceral/te<br>Incarcerat/ed/es/ion<br>Jail/ed/s<br>Prison/er/s<br>Detention<br>Detainee/d/s<br>Corrections<br>Correctional<br>Parole/e/s<br>Probation<br>Offender/s<br>Crime/s<br>Criminal/s/ized/ization<br>Criminol/olgy/ogical<br>"Justice Involvement"<br>"Justice Involved"<br>Institutionaliz/ation/ed<br>"restorative justice"<br>Inmate/s<br>Felon/s/y<br>Misdemeanor/s<br>Convict/s/ed<br>Imprison/ed<br>Post-Release<br>"Justice System"	"Prisons"[Mesh]<br>"Prisoners"[Mesh]<br>"Criminals"[Mesh]<br>"Crime Victims"[Mesh]<br>"Criminology"[Mesh]	**("Prisons"[Mesh] OR "Prisoners"[Mesh] OR "Criminals"[Mesh] OR "Crime Victims"[Mesh] OR "Criminology"[Mesh] OR "Institutionalization"[Mesh]) OR (prison*[Title/Abstract] OR "justice system*"[Title/Abstract] OR incarcerat*[Title/Abstract] OR carcera*[Title/Abstract] OR institutionaliz*[Title/Abstract] OR criminal*[Title/Abstract] OR criminol*[Title/Abstract] OR crime*[Title/Abstract] OR "restorative justice"[Title/Abstract] OR corrections[Title/Abstract] OR correctional[Title/Abstract] OR inmate*[Title/Abstract] OR felon*[Title/Abstract] OR misdemeanor*[Title/Abstract] OR convict*[Title/Abstract] OR imprison*[Title/Abstract] OR jail*[Title/Abstract] OR offender*[Title/Abstract] OR**

© 2024 Jolin JR et al. *JAMA Health Forum*.

			**probation[Title/Abstract] OR parole*[Title/Abstract] OR detention[Title/Abstract] OR detained[Title/Abstract] OR "post-release"[Title/Abstract] OR "justice involved"[tiab:~4] OR "justice involvement"[Tiab:~3])**

© 2024 Jolin JR et al. *JAMA Health Forum*.

eTable 3. Search Strategy and Results, Conducted December 31, 2023

DATABASE (PLATFORM)	SEARCH	RESULTS
**PUBMED (NATIONAL LIBRARY OF MEDICINE)**	("Prisons"[Mesh] OR "Prisoners"[Mesh] OR "Criminals"[Mesh] OR "Crime Victims"[Mesh] OR "Criminology"[Mesh] OR "Institutionalization"[Mesh] OR prison*[Title/Abstract] OR "justice system*"[Title/Abstract] OR incarcerat*[Title/Abstract] OR carcera*[Title/Abstract] OR institutionaliz*[Title/Abstract] OR criminal*[Title/Abstract] OR criminol*[Title/Abstract] OR crime*[Title/Abstract] OR "restorative justice"[Title/Abstract] OR corrections[Title/Abstract] OR correctional[Title/Abstract] OR inmate*[Title/Abstract] OR felon*[Title/Abstract] OR misdemeanor*[Title/Abstract] OR convict*[Title/Abstract] OR imprison*[Title/Abstract] OR jail*[Title/Abstract] OR offender*[Title/Abstract] OR probation[Title/Abstract] OR parole*[Title/Abstract] OR detention[Title/Abstract] OR detained[Title/Abstract] OR "post-release"[Title/Abstract] OR "justice involved"[tiab:~4] OR "justice involvement"[Tiab:~3]) AND ("Patient Protection and Affordable Care Act"[Mesh] OR "Medicaid"[Mesh] OR "111-148"[Title/Abstract] OR "PL111-148"[Title/Abstract] OR "affordable care act*"[Title/Abstract] OR ACA[Title/Abstract] OR "health care reform*"[Title/Abstract] OR obamacare[Title/Abstract] OR "obama care"[Title/Abstract] OR "patient protection act*"[Title/Abstract] or "section 1115" [tiab] OR "dependent coverage"[tiab] OR "medicaid"[Title/Abstract])	646
**CINAHL COMPLETE (EBSCO)**	(MH ("Correctional Health Services" OR "Prisoners" OR "Correctional Facilities" OR "Public Offenders+" OR "Institutionalization" OR "Deinstitutionalization" OR "Criminal Justice" OR "Crime") OR TI (prison* OR "justice system*" OR incarcerat* OR carcera* OR institutionaliz* OR criminal* OR criminol* OR crime* OR "restorative justice" OR corrections OR correctional OR inmate* OR felon* OR misdemeanor* OR convict* OR imprison* OR jail* OR offender* OR probation OR parole* OR detention OR detained OR "post-release" OR (justice N4 involv*)) OR AB (prison* OR "justice system*" OR incarcerat* OR carcera* OR institutionaliz* OR criminal* OR criminol* OR crime* OR "restorative justice" OR corrections OR correctional OR inmate* OR felon* OR misdemeanor* OR convict* OR imprison* OR jail* OR offender* OR probation OR parole* OR detention OR detained OR "post-release" OR (justice N4 involv*))) AND (MH ("Patient Protection and Affordable Care Act+" OR "Medicaid+") OR TI ("111-148" OR "PL111-148" OR "affordable care act*" OR ACA OR "health care reform*" OR obamacare OR "obama care" OR "patient protection act*" or "section 1115" OR "dependent coverage" OR medicaid) OR AB ("111-148" OR "PL111-148" OR "affordable care act*" OR ACA OR "health care reform*" OR obamacare OR "obama care" OR "patient protection act*" or "section 1115" OR "dependent coverage" OR medicaid))	481

© 2024 Jolin JR et al. *JAMA Health Forum*.

**APA PSYCINFO (EBSCO)**	(DE ("Criminal Justice" OR "Criminal Conviction" OR "Exoneration" OR "Juvenile Justice" OR "Restorative Justice" OR "Distributive Justice" OR "Juvenile Justice" OR "Diversion Programs" OR "Correctional Institutions" OR "Prisons" OR "Reformatories" OR "Halfway Houses" OR "Incarceration" OR "Rehabilitation" OR "Formerly Incarcerated" OR "Incarcerated" OR "Reintegration" OR "Probation" OR "Prisoner Abuse" OR "Parole" OR "Criminal Record") OR TI (prison* OR "justice system*" OR incarcerat* OR carcera* OR institutionaliz* OR criminal* OR criminol* OR crime* OR "restorative justice" OR corrections OR correctional OR inmate* OR felon* OR misdemeanor* OR convict* OR imprison* OR jail* OR offender* OR probation OR parole* OR detention OR detained OR "post-release" OR (justice N4 involv*))<br>OR AB (prison* OR "justice system*" OR incarcerat* OR carcera* OR institutionaliz* OR criminal* OR criminol* OR crime* OR "restorative justice" OR corrections OR correctional OR inmate* OR felon* OR misdemeanor* OR convict* OR imprison* OR jail* OR offender* OR probation OR parole* OR detention OR detained OR "post-release" OR (justice N4 involv*))) AND (DE ("Affordable Care Act" OR "Medicaid") OR TI ("111-148" OR "PL111-148" OR "affordable care act*" OR ACA OR "health care reform*" OR obamacare OR "obama care" OR "patient protection act*" or "section 1115" OR "dependent coverage" OR medicaid) OR AB ("111-148" OR "PL111-148" OR "affordable care act*" OR ACA OR "health care reform*" OR obamacare OR "obama care" OR "patient protection act*" or "section 1115" OR "dependent coverage" OR medicaid))	270
**EMBASE (ELSEVIER)**	1. 'correctional health care'/exp OR 'correctional facility'/exp OR 'prisoner'/exp OR 'offender'/exp OR 'crime'/de OR 'punishment'/exp OR 'criminal justice'/exp OR (prison* OR 'justice system*' OR incarcerat* OR carcera* OR institutionaliz* OR criminal* OR criminol* OR crime* OR 'restorative justice' OR corrections OR correctional OR inmate* OR felon* OR misdemeanor* OR convict* OR imprison* OR jail* OR offender* OR probation OR parole* OR detention OR detained OR post-release OR (justice NEAR/2 involv*)):ti,ab,kw<br><br>2. 'medicaid'/exp/mj OR 'health care policy'/exp OR ('111-148' OR 'PL111-148' OR 'affordable care act*' OR ACA OR 'health care reform*' OR obamacare OR 'obama care' OR 'patient protection act*' or 'section 1115' OR (coverage NEAR/2 (dependent* OR expand*)) OR medicaid):ti,ab,kw<br><br>3. #1 AND #2 AND [embase]/lim AND [2014-2023]/py	2158
**SOCIAL SCIENCE DATABASE (PROQUEST)**	((MAINSUBJECT.EXACT("Criminals") OR MAINSUBJECT.EXACT("Institutionalism") OR MAINSUBJECT.EXACT("Criminal justice") OR MAINSUBJECT.EXACT("Prison reform") OR MAINSUBJECT.EXACT("Institutionalization") OR MAINSUBJECT.EXACT("Prisoners") OR	555

© 2024 Jolin JR et al. *JAMA Health Forum*.

	MAINSUBJECT.EXACT("Imprisonment") OR MAINSUBJECT.EXACT("Prisons")) OR noft((prison* OR "justice system*" OR incarcerat* OR carcera* OR institutionaliz* OR criminal* OR criminol* OR crime* OR "restorative justice" OR corrections OR correctional OR inmate* OR felon* OR misdemeanor* OR convict* OR imprison* OR jail* OR offender* OR probation OR parole* OR detention OR detained OR "post-release" OR (justice N4 involv*)))) AND (MAINSUBJECT.EXACT("Patient Protection & Affordable Care Act 2010-US") OR noft("111-148" OR "PL111-148" OR "affordable care act*" OR ACA OR "health care reform*" OR obamacare OR "obama care" OR "patient protection act*" OR "section 1115" OR "dependent coverage" OR medicaid))	
**WEB OF SCIENCE (CLARIVATE)**	((TI=("111-148" OR "PL111-148" OR "affordable care act*" OR ACA OR "health care reform*" OR obamacare OR "obama care" OR "patient protection act*" or "section 1115" OR "dependent coverage" OR medicaid)) OR AB=("111-148" OR "PL111-148" OR "affordable care act*" OR ACA OR "health care reform*" OR obamacare OR "obama care" OR "patient protection act*" or "section 1115" OR "dependent coverage" OR medicaid)) OR AK=("111-148" OR "PL111-148" OR "affordable care act*" OR ACA OR "health care reform*" OR obamacare OR "obama care" OR "patient protection act*" or "section 1115" OR "dependent coverage" OR medicaid) AND ((TI=((prison* OR "justice system*" OR incarcerat* OR carcera* OR institutionaliz* OR criminal* OR criminol* OR crime* OR "restorative justice" OR corrections OR correctional OR inmate* OR felon* OR misdemeanor* OR convict* OR imprison* OR jail* OR offender* OR probation OR parole* OR detention OR detained OR "post-release" OR (justice NEAR/4 involv*)))) OR AB=((prison* OR "justice system*" OR incarcerat* OR carcera* OR institutionaliz* OR criminal* OR criminol* OR crime* OR "restorative justice" OR corrections OR correctional OR inmate* OR felon* OR misdemeanor* OR convict* OR imprison* OR jail* OR offender* OR probation OR parole* OR detention OR detained OR "post-release" OR (justice NEAR/4 involv*)))) OR AK=((prison* OR "justice system*" OR incarcerat* OR carcera* OR institutionaliz* OR criminal* OR criminol* OR crime* OR "restorative justice" OR corrections OR correctional OR inmate* OR felon* OR misdemeanor* OR convict* OR imprison* OR jail* OR offender* OR probation OR parole* OR detention OR detained OR "post-release" OR (justice NEAR/4 involv*)))	420
		4530

© 2024 Jolin JR et al. *JAMA Health Forum*.

eTable 4. Description of Included Studies, Including Provision of Patient Protection and Affordable Care Act (ACA) Analyzed, Data Source, Study Period, Sample, Design, and Outcomes

Source	Provision of ACA	Data source	Study period	Age group	Definition of criminal justice involvement	Population analyzed[a]	Individuals, No.[a]	Study design	Outcomes for people with criminal legal involvement[b]	Association, significance, point estimate, and uncertainty[c]
Winkelman et al,[1] 2020	Medicaid expansion	TEDS-A	1992-2017	Adults and minors	Whether a pregnant woman with OUD was referred to by criminal justice agencies. Criminal justice includes referral from police, probation officers, judges, prosecutors, DUI/DWI court, or parole board.	Pregnant women with OUD referred to medication for OUD treatment by criminal justice agencies in dataset	17 563	Serial cross-sectional analysis; multiple logistic regression and DiD	The extent to which medications for OUD changed among pregnant women with a criminal justice referral with OUD in states that expanded Medicaid	Positive, significant, adjusted DiD estimate: 12.0 pp (95% CI, 1.0-23.0)
									The extent to which pregnant women referred by criminal justice agencies were more or less likely to have received medications for OUD in states that expanded Medicaid compared with women in states that did not expand Medicaid	Positive, significant, adjusted DiD estimate: 27.2 pp (95% CI, 11.3-43)
Winkelman et al,[2] 2016	ACA broadly	NSDUH	2008-2015	Adults	Those who reported being arrested and booked (excluding minor traffic violations), paroled, or on probation in the 12 mo preceding the	Adults aged 19-64 y between 2008 and 2014 with a history of justice involvement during the prior 12 mo in survey	15 899	Serial, pooled cross-sectional analysis; DiD	Association between the dependent coverage provision and uninsurance rates	Negative, significant, DiD estimate: −13.0% (95% CI, −18.8 to −7.2)
									Association between the dependent coverage provision	Positive, significant, DiD estimate:

© 2024 Jolin JR et al. *JAMA Health Forum*.

					survey interview date				and private insurance rates	13.1% (95% CI, 7.6-18.7)
									Association between the dependent coverage provision and Medicaid coverage rates	Positive, insignificant, DiD estimate: 0.7% (95% CI, −3.5 to 4.9)
									Association between Medicaid expansion and Marketplace plans in 2014 and uninsurance rates	Negative, significant, estimate not reported in main text
									Association between Medicaid expansion/Marketplace plans in 2014 and Medicaid enrollment	Positive, significant, DiD estimate: 6.3% (*P* < .001)
									Medicaid and private insurance association with rates of treatment among criminal legal–involved individuals with serious mental illness compared with those without insurance	Positive, significant, ATE estimate: 24.3% (*P* < .001); positive, significant, ATE estimate: 14.8% (*P* < .05), respectively
									Among criminal legal–involved individuals with alcohol use or alcohol dependence, Medicaid and private insurance association with rates of treatment	Positive, significant, ATE estimate: 6.6% (*P* < .05); positive, significant, ATE estimate: 6.0% (*P* <

© 2024 Jolin JR et al. *JAMA Health Forum*.

										.05), respectively
									Association between Medicaid alone and SUD treatment among those with illicit drug use or dependence	Positive, significant, ATE estimate: 12.6% (*P* < .05)
Winkelman et al,[3] 2017	ACA, broadly	National Survey of Family Growth	2008-2015	Adults	In the last 12 mo, whether respondent spent any time in a jail, prison, or a juvenile detention facility and whether they ever spent time in a jail, prison, or a juvenile detention center	Men aged 18-44 y with a history of incarceration in survey	3476	Serial cross-sectional analyses, logistic regression, and multivariable logistic regression	Association between ACA and uninsurance rate	Negative, significant, estimate: 5.9 pp (95% CI, −11.5 to −0.4)
									Association between ACA and private insurance rate	Positive, significant, estimate: 6.8 pp (95% CI, 0.1-13.3)
									Association between ACA and public insurance rate	Negative, insignificant, estimate: −0.8 pp (95% CI, −6.3 to 4.6)
									Association between ACA and gaps in insurance coverage	No change: pre-ACA, 45.5%; post-ACA, 45.6% (*P* = .97)
									Difference in reporting regular source of primary care after ACA implementation, relative to men without history of incarceration	Significantly lower estimated difference: −13.0 pp (95% CI, −18.3 to −7.6)
									Reporting using emergency department as regular source of care or for any reason after ACA expansion, compared	Significantly more likely, estimated difference: 2.7 pp (95% CI, 0.7-4.7);

© 2024 Jolin JR et al. *JAMA Health Forum*.

									with never incarcerated	significantly more likely, estimated difference: 6.0 pp (95% CI, 3.0-9.0), respectively
Sledge et al,[4] 2022	Medicaid expansion	TEDS, data on state Medicaid expansion and data on drug-related deaths from the CDC	2000-2017	Unclea r	"The criminal justice-referred admissions analyzed included referrals from judges, prosecutors, probation officers, and others affiliated with federal, state, or county court systems."[4]	"TEDS captures individual admissions, rather than individual people. This means that, throughout, our unit of analysis is individual admissions to substance use treatment facilities."[4]	339 427 Individual admissions referred to by criminal justice agency	Serial cross-sectional analysis and logistic regression	Odds of treatment for admissions in states with Medicaid expansion	Positive, significant, odds ratio: 3.01 (95% CI, 2.99-3.04)
									Odds of MAT in Medicaid expansion states for criminal justice–referred admission	Negative, significant, odds ratio: 0.13 (95% CI, 0.12-0.13)
									Odds of treatment for admissions in states with Medicaid expansion, for American Indian, Asian, Black, Hispanic, and other race individuals relative to non-Hispanic White individuals	Greater likelihood of being assigned MAT in expansion states, but less so compared with White counterparts; exact estimates vary
									Odds of MAT in Medicaid expansion states for criminal justice–referred admission for Black individuals relative to White individuals	Significantly lower, odds ratio: 0.64 (95% CI, 0.61-0.68)
									Odds of MAT in Medicaid expansion states for criminal justice–referred	Significantly lower, odds ratio: 0.559

© 2024 Jolin JR et al. *JAMA Health Forum*.

									admission for Hispanic individuals relative to White individuals	(95% CI, 0.54-0.58)
Simes and Jahn,[5] 2022	Medicaid expansion	FBI UCR program data, US Census Bureau Population Estimates Program intercensal county population data, Robert Wood Johnson Foundation County Health Rankings and Roadmaps and the US Census Bureau American Community Survey 5-y estimates, National Center for Health Statistics Urban-Rural Classification, Census Bureau Annual Survey of State and Local Government Finances, CDC WONDER	2011-2016	Unclea r	All arrests, drug arrests, violent arrests, and low-level arrests	US county	3035	DiD	Rate of arrest in counties in Medicaid expansion states compared with counties in non-expansion states	Significant 20%-32% negative difference (*P* < .01)
									Difference in arrests for violent arrests in all 3 years of Medicaid expansion	Significant 19%-29% negative difference (*P* < .01)
									Difference in low-level arrests between expansion states compared with non-expansion state	Significant 24%-28% negative difference (*P* < .01)
									County-level drug-related arrests in expansion counties relative to counties in non-expansion states	Significant 25%-41% negative difference (*P* < .01)

© 2024 Jolin JR et al. *JAMA Health Forum*.

Saloner et al,[6] 2016	ACA, broadly	NSDUH	2004-2014	Adults	"Individuals were defined as having prior-year involvement with the criminal justice system if they reported being either arrested and booked or on probation or parole in the prior twelve months."[6]	Individuals reporting being either arrested and booked or on probation or parole in the prior 12 mo with SUD who responded to the survey	11.939	Before-and-after design	Uninsurance rate	Negative association, 27.7%-38%
									Medicaid enrollment	Positive association, 20.8%-29.5%
									Medicare enrollment	Positive association, 5.1%-9.9%
									Share of individuals with SUD and criminal justice involvement receiving SUD treatment (in any setting)	No change, 30%-33%
									Change in treatment setting among those with SUD who received treatment	Suggestive but not all were statistically significant; estimates vary
									Source of payment for treatment being Medicaid	Significant positive association, 19.5% in 2009-2013 to 29% in 2014 ($P < .05$)
Rosen et al,[7] 2016	Medicaid expansion	Survey of Inmates in State Correctional Facilities	2004	Adults	Male state prisoners aged 18-64 y	Male prisoners aged 18-64 y with a chronic health condition in survey	643 290	Projection of number of male prisoners with a chronic condition who would be eligible for health care coverage at	Projected proportion of prisoners earning ≥400% FPL and thus would be ineligible for Medicaid or tax credits	20% and 17%, respectively
									In expansion states, projected proportion of prisoners qualifying for Medicaid or tax credits	73%

© 2024 Jolin JR et al. *JAMA Health Forum*.

								release using weighted 2004 survey data	In nonexpansion states, proportion of prisoners qualified for tax credits but were projected to have incomes 100% FPL and would not qualify for any support (Figure 1[7]), placing them in the coverage gap	22%
									In nonexpansion states, proportion of prisoners qualified for tax credits	54%
Knapp et al,[8] 2019	ACA, broadly	NSDUH	2008-2016	Adults	"Individuals on probation were identified as such if they reported being on probation in the past year."[8]	Individual respondents reporting being on probation in the past y	11 511 (Unweighted)	Before-and-after design	Change in insurance coverage rates between 2013 and 2016	Positive, significant association: 7.4 pp (95% CI, 11.8-23.0)
									Changes in private health insurance, other insurance coverage rates between 2013 and 2017	Not significant; no estimate provided
									Changes in Medicaid insurance coverage rates between 2013 and 2017	Positive, significant association: 18.7%-31.3% ($P <$ .001)
Khatri et al,[9] 2021	Medicaid expansion	TEDS-A	2008-2017	Adults	Referred to substance use treatment by criminal justice entity. Criminal justice referrals included referrals from the police, probation officers, judges,	Adults with OUD referred by a criminal justice agency in dataset	674 744	Serial cross-sectional analysis and DiD	Among individuals referred by criminal justice agencies, rates of medications for OUD receipt among those in Medicaid expansion states relative those in non-expansion states	Higher, significant association: ARR, 2.07 (95% CI, 2.00-2.13)
									Between 2008-2013 and 2015-2017,	Positive, significant,

© 2024 Jolin JR et al. *JAMA Health Forum*.

					prosecutors, driving under the influence/driving while intoxicated courts, and parole boards				change in receipt of medications for OUD among individuals referred by the criminal justice agencies in states that expanded Medicaid compared with such individuals in states that did not expand Medicaid	adjusted DID estimate: 8.6 pp (95% CI, 2.2-15.0)
									Adjusted rates of medications for OUD for individuals in the US referred to treatment in Medicaid expansion and non-expansion states, by treatment referral source, 2008-2017, among non-Hispanic White, non-Hispanic Black, Hispanic, American Indian and Alaska Native, and other race populations	Positive significant DiD estimate: 9.8 (*P* < .01); positive significant DiD estimate: 6.5 (*P* < .05); positive insignificant DiD estimate: 5.4 (*P* > .05); positive significant DiD estimate: 5.9 (*P* < .01); positive, insignificant DiD estimate: 9.1 (*P* > .05), respectively
									Adjusted rates of medications for OUD for individuals in the US referred to treatment in Medicaid expansion and nonexpansion states, by treatment referral	Positive, significant DiD estimate: 9.5 (*P* < .01); positive, insignificant DiD

© 2024 Jolin JR et al. *JAMA Health Forum*.

									source, 2008-2017, in ambulatory, residential settings	estimate: 5.9 (*P* > .05), respectively
Howell et al,[10] 2019	ACA, broadly	NSDUH	2011-2017	Adults	"We determined criminal justice involvement in the past year if respondents reported being arrested and booked in the past year (excluding minor traffic violations) or under community supervision via probation or parole in the past year."[10]	Individuals who reported criminal justice involvement in the past y in survey	14 044	Before-and-after design; multivariate logistic regression	Proportion of individuals who had insurance coverage among those who reported criminal justice involvement in the past y	Positive, significant association: 13.4 pp (95% CI, 8.5-18.3), from 61.0% (95% CI, 56.7-65.2) in 2011-2013 to 74.4% (95% CI, 71.8-77.1) in 2014-2017
									Proportion of individuals with criminal justice involvement in the past y who had Medicaid insurance	Positive, significant association, 25.4%-37.4%, a difference of 12.0 pp (95% CI, 7.2-16.7)
									Proportion of individuals with criminal justice involvement who had private insurance	Positive, significant association, 24.0%-28.7%, a difference of 4.7 pp (95% CI, 0.5-8.9)
									Among adults with past-year criminal justice involvement, change in receipt of any mental health treatment following ACA implementation	Negative, insignificant association, from 50.7% (95% CI, 47.3-54.2) in 2011-2013 to 47.3% (95% CI, 44.0-50.6) in

© 2024 Jolin JR et al. *JAMA Health Forum*.

										2014-2017, a difference of −3.4 pp (95% CI, −8.0 to 1.1)
									Among individuals with criminal justice involvement in the past y, change in the proportion reporting any inpatient mental health treatment, outpatient mental health treatment, receipt of prescription medication for a mental disorder	Negative, insignificant association: inpatient mental health treatment (−2.5 pp; 95% CI, −5.7 to 0.5), outpatient mental health treatment (−2.0 pp; 95% CI, −6.2 to 2.2), or receipt of prescription medication for a mental disorder (−4.6 pp; 95% CI, −9.0 to 0.2)
									Change in the proportion of individuals with unmet mental health care needs following ACA implementation among individuals with criminal justice involvement in the past y	Negative, insignificant association: 33.1% in 2011-2013 and 32.2% in 2014-2017, a difference of −0.8 pp (95% CI, −5.4 to 3.6)
									Among people with criminal legal	Negative, insignificant

© 2024 Jolin JR et al. *JAMA Health Forum*.

									involvement who reported an unmet mental health need, change in those reporting that they did not get care because of financial reasons	association: 61.3% in 2011-2013 and 49.0% in 2014-2017, a difference of −12.3 pp (95% CI, −4.4 to −20.1)
									Among people with criminal legal involvement, change in those reporting that Medicaid paid for their mental health treatment	Positive, insignificant association, from 25.3% to 29.9% (a difference of 4.5 pp; 95% CI, −3.4 to 12.5)
									Among people with criminal legal involvement, change in the proportion that reported their mental health treatment was paid for by themselves or their family members	Negative, insignificant association, from 26.8% to 23.4% (a difference of −3.4 pp; 95% CI, −10.0 to 3.3)
Howell et al,[11] 2022	Medicaid expansion	NSDUH	2010-2017	Adults	"We defined presence of past year criminal legal involvement based on responses to questions regarding past year arrest (excluding minor traffic violations) and being on community	Adults aged 18-64 y who reported (1) a household income of ≤138% of the federal poverty level and (2) past-year criminal legal involvement	9910	DiD	Insurance coverage	Significant positive association, 14.9 pp (95% CI, 5.4-24.3)
									Medicaid coverage	Significant positive association, 19.1 pp (95% CI, 10.2-28.0)
									Private insurance coverage	No significant change, −1.1

© 2024 Jolin JR et al. *JAMA Health Forum*.

					correctional supervision (parole or probation) in the past year."[11]					pp (95% CI, −5.9 to 3.6)
									Other forms of coverage, Medicare, Tricare, or VHA	No significant change, 1.5 pp (95% CI, −1.5 to 4.5)
He and Barkowski,[12] 2020	Medicaid expansion	FBI UCR program data, Interuniversity Consortium for Political and Social Research	2010-2016; 2010-2014; 2016	Unclear	Commission of property crime (burglary, larceny theft, motor vehicle theft), commission of violent crime (robbery, aggravated assault)	All states and Washington, DC, and counties on Medicaid expansion state borders	51 and 541, Respectively	DiD	Natural log of property crime rate per 100 000 residents	Negative, insignificant association, −0.019 (SE, 0.020)
									Natural log of burglary crime rate per 100 000 residents	Negative, significant association, −0.038 (SE, 0.022)
									Natural log of larceny theft crime rate per 100 000 residents	Negative, insignificant association, −0.005 (SE, 0.019)
									Natural log of motor vehicle crime rate per 100 000 residents	Negative, significant association, −0.121 (SE, 0.039)
									Natural log of violent crime rate per 100 000 residents	Negative, significant association, −0.049 (SE, 0.016)
									Natural log of criminal homicide crime rate per 100 000 residents	Negative, significant association, −0.112 (SE, 0.036)
									Natural log of robbery crime rate per 100 000 residents	Negative, significant association, −0.089 (SE, 0.029)
									Natural log of aggravated assault	Negative, insignificant

© 2024 Jolin JR et al. *JAMA Health Forum*.

									crime rate per 100 000 residents	association, −0.022 (SE, 0.017)
Gutierrez and Pettit,[13] 2020	ACA, broadly	NSDUH	2009-2016	Adults	"Individuals were identified as recently incarcerated if they reported being on probation or parole in the previous 12 months."[13]	Recently incarcerated men aged 18-64 y surveyed	7157	DiD	Health uninsurance rate	Negative, significant association, −15.4 (95% CI, −18.8 to −12.2)
									Hospital stay past 12 mo	Negative, significant association, −3.0 (95% CI, −5.2 to −0.1)
									Emergency department visit past 12 mo	Negative, insignificant association, −3.1 (95% CI, −7.0 to 0.8)
									Diabetes	Positive, significant association, 0.7 (95% CI, −0.6 to 1.9)
									Hypertension	Negative, significant association, −3.5 (95% CI, −5.7 to −1.1)
									Mental illness	Positive, insignificant association, 1.6 (95% CI, −2.0 to 5.2)
									SUD	Negative, insignificant association, −1.7 (95% CI, −5.3 to 5.4)

© 2024 Jolin JR et al. *JAMA Health Forum*.

Fry et al,[14] 2020	Medicaid expansion	48 Continuous mo of individual-level booking and release dates from 6 urban county jails	4 y Total (years varied for state pairs)	Unclea r	Rearrest after an individual has been arrested once	6 Urban counties in the Midwest, Southwest, and Southeast	6	CITS, DiD	Mean change in probability of rearrest in Midwest treatment-control county pair	Negative association, 1.49 pp
									Mean change in probability of rearrest in Southwest treatment-control county pair	Negative association, 3.6 pp
									Mean change in probability of rearrest in Southeast treatment-control county pair	Positive association, 1.61 pp
Dickson et al,[15] 2018	ACA, broadly	Complete responses from baseline and the 3-mo follow-up interviews from 3 rural jails in 3 different Appalachian counties	Novembe r 2012-August 2015	Adults	Substance-involved women in rural Appalachian Kentucky who were released into the community after incarceration	Substance-involved women in rural Appalachian Kentucky who were released into the community after incarceratio n who completed baseline and 3-mo follow-up and had complete data	371	Bivariate analysis and multivariate logistic regression	Days bothered by health problems during 3-mo follow-up period	Positive, insignificant association, 0.5 d on average
									Percentage insured for ≥1 mo during 3-mo follow-up period	Positive, significant association, 57 pp on average
									Percentage have usual source of nonemergency, outpatient health care	Positive, significant association, 11 pp on average
									Percentage currently being treated for a health problems	Positive, insignificant association, 4.1 pp on average
									Percentage who went to emergency department for health problems during the 3-mo follow-up period	Negative, significant association, −8.7 pp on average
									Percentage who went to substance use treatment program	Negative, insignificant association,

© 2024 Jolin JR et al. *JAMA Health Forum*.

									during the 3-mo follow-up period	−1.8 pp on average
									Availability of services (1, not at all available; 10, extremely available)	Positive, insignificant association, 0.5 units
									Having a usual source of nonemergency, outpatient care at 3-mo follow- up	Negative, insignificant association, 0.53 (95% CI, 0.27-1.04)
Aslim et al,[16] 2022	Medicaid expansion	National Corrections Reporting Program	2010-2016	Adults	Individuals sentenced to prison in the US but not jails	Individuals who committed violent, property, drug, or public order crimes who recidivated at 1 and 2 y (8 samples)	248 410 (Violent, 1 y), 250 032 (property, 1 y), 255 295 (drug, 1 y), 161 262 (public order, 1 y), 209 961 (violent, 2 y), 213 726 (property, 2 y), 218 634 (drug, 2 y), 137 603 (public order, 2 y	DiD	Whether inmate returns to prison within 1 y of release	Significantly reduced, estimates vary
									Whether inmate returns to prison within 2 y of release	Significantly reduced, estimates vary
									Count of admissions to SUD treatment, aggregated on the state level	Significant positive association, estimates vary
Howell et al,[17] 2023	Medicaid expansion	NSDUH	2010-2017	Adults	"Defined past-year criminal legal involvement on the basis of responses to questions regarding past-year arrest (excluding minor traffic violations), parole, or probation."[17]	Individuals aged 18-64 y with a household income ≤138% of the federal poverty level, with past-year criminal legal involvement , and who	9910	DiD	Proportion of sample with SUDs reporting being enrolled in a private or public health insurance plan	Positive, significant association, 18 pp (P = .04)
									Proportion of sample with other mental health disorder reporting being enrolled in a private or public health insurance plan	Positive, significant association, 16 pp (P = .04)
									Proportion of sample with SUDs reporting	Negative, insignificant

© 2024 Jolin JR et al. *JAMA Health Forum*.

						met diagnostic criteria for a past-year SUD or any other mental disorder			receipt of inpatient or outpatient treatment for illicit drug or alcohol use in the past year	association, −0.7 pp (P = .96)
									Proportion of sample with other mental health disorders reporting receipt of inpatient, outpatient, or pharmacy services for mental health in the past y	Positive, significant association, 8 pp (P = .18)
Testa and Porter,[18] 2023	Medicaid expansion	National Longitudinal Study of Adolescent to Adult Health	1994-1995 (wave I); 2008-2009 (wave IV); 2016-2018 (wave V)	Adults and minors	"Have you ever served time in a jail, prison, juvenile detention center, or other correctional facility? (1=yes, 0=no)."[18]	Individuals aged 12-19 y (wave I); individuals aged 24-32 y (wave IV); individuals aged 33-43 y (wave V)	8965	Panel survey; multiple logistic regression	Odds of being insured, for those with a history of being incarcerated	Positive, insignificant association, odds ratio: 1.630 (95% CI, 0.861-3.089)
									Odds of being insured under public insurance program, for those with a history of being incarcerated	Positive, significant association, odds ratio: 2.402 (95% CI, 1.257-4.588)
									Odds of being insured, for those with a history of being incarcerated among White respondents	Positive, insignificant association, odds ratio: 2.055 (95% CI, 0.910-4.640)
									Odds of being insured, for those with a history of being incarcerated among Black respondents	Positive, insignificant association, odds ratio: 1.848 (95% CI, 0.430-7.938)
									Odds of being insured, for those with a history of being	Negative, insignificant association,

© 2024 Jolin JR et al. *JAMA Health Forum*.

									incarcerated among Hispanic respondents	odds ratio: 0.936 (95% CI, 0.193-4.540)
									Odds of being insured under public insurance program, for those with a history of being incarcerated among White respondents	Positive, significant association, odds ratio: 4.599 (95% CI, 1.818-11.637)
									Odds of being insured under public insurance program, for those with a history of being incarcerated among Black respondents	Positive, insignificant association, odds ratio: 1.417 (95% CI, 0.456-4.402)
									Odds of being insured under public insurance program, for those with a history of being incarcerated among Hispanic respondents	Positive, insignificant association, odds ratio: 1.564 (95% CI, 0.254-9.646)
Fone et al,[19] 2023	ACA Dependent Coverage Mandate	FBI National Incident-Based Reporting System	2008-2013	Adults aged 22-25 y and 27-29 y	"[C]riminal incidents leading to an arrest for part I property crimes (larceny, motor vehicle theft, burglary, or arson), part I violent crimes (aggravated assault, robbery, rape, or murder), and all crimes (property and violent crime) for those ages 22-25 and 27-29."[19]	Law enforcement agency	Varies depending on model specification; smallest No. for main estimate is 1 157 688; largest No. for main estimate is 1 663 354	DiD	Number of property crime arrests involving individuals aged 22-25 y during the enactment period	Estimates and SEs vary depending on model specification; all show a negative association, but not all are statistically significant
									Number of property crime arrests involving individuals aged 22-25 y in the postimplementation period	Estimates and SEs vary depending on model specification

© 2024 Jolin JR et al. *JAMA Health Forum*.

										; all show a negative association, and all are statistically significant at 5% level
									Number of violent crime arrests involving individuals aged 22-25 y during the enactment period	Estimates and SEs vary depending on model specification; all show a negative association, but none are statistically significant
									Number of violent crime arrests involving individuals aged 22-25 y in the postimplementation period	Estimates and SEs vary depending on model specification; all show a positive association, but none are statistically significant

Abbreviations: ARR, adjusted risk ratio; ATE, average treatment effect; CDC, Centers for Disease Control and Prevention; CITS, comparative interrupted time series; DiD, difference-in-differences; DUI, driving under the influence; DWI, driving while intoxicated; FBI, Federal Bureau of Investigation; FPL, federal poverty level; MAT, medication-assisted treatment; NSDUH, National Survey on Drug Use and Health; OUD, opioid use disorder; pp, percentage point; SUD, substance use disorder; TEDS, Treatment Episode Data Set; TEDS-A; Treatment Episode Data Set–Admissions; UCR, Uniform Crime Reporting; VHA, Veterans Health Administration; WONDER, Wide-Ranging Online Data for Epidemiologic Research.

[a]Because our review is interested principally in people with criminal legal involvement, we report units of analysis and their corresponding numeric values as they relate to analyses for people with criminal legal involvement.

[b]Outcomes in this table are not necessarily exhaustive of all outcomes assessed in included studies because we only report those outcomes explicitly for criminal legal–involved populations and that fit within our 5 primary areas of outcomes defined prior to initiating this study: insurance coverage rates, access to care, cost of care, health outcomes, and social welfare outcomes.

[c]Wherever possible, this column reports the direction and significance level as reported in the primary analyses included in the main text of the articles (ie, we do not report results as they may appear in appendices or sensitivity analyses). Moreover, we include adjusted and/or weighted point estimates and corresponding uncertainty statistics as reported in the main text of the included articles.

© 2024 Jolin JR et al. *JAMA Health Forum*.

eReferences.

1. Winkelman TNA, Ford BR, Shlafer RJ, McWilliams A, Admon LK, Patrick SW. Medications for opioid use disorder among pregnant women referred by criminal justice agencies before and after Medicaid expansion: a retrospective study of admissions to treatment centers in the United States. PLoS Med. 2020;17(5):e1003119. Medline:32421717 doi:10.1371/journal.pmed.1003119
2. Winkelman TN, Kieffer EC, Goold SD, Morenoff JD, Cross K, Ayanian JZ. Health insurance trends and access to behavioral healthcare among justice-involved individuals—United States, 2008-2014. J Gen Intern Med. 2016;31(12):1523-1529. Medline:27638837 doi:10.1007/s11606-016-3845-5
3. Winkelman TN, Choi H, Davis MM. The Affordable Care Act, insurance coverage, and health care utilization of previously incarcerated young men: 2008-2015. Am J Public Health. 2017;107(5):807-811. Medline:28323472 doi:10.2105/AJPH.2017.303703
4. Sledge D, Thomas HF, Hoang BL, Mohler G. Impact of Medicaid, race/ethnicity, and criminal justice referral on opioid use disorder treatment. J Am Acad Psychiatry Law. 2022;50(4):545-551. Medline:36167412 doi:10.29158/jaapl.210137-21
5. Simes JT, Jahn JL. The consequences of Medicaid expansion under the Affordable Care Act for police arrests. PLoS One. 2022;17(1):e0261512. Medline:35020737 doi:10.1371/journal.pone.0261512
6. Saloner B, Bandara SN, McGinty EE, Barry CL. Justice-involved adults with substance use disorders: coverage increased but rates of treatment did not in 2014. Health Aff (Millwood). 2016;35(6):1058-1066. Medline:27269023 doi:10.1377/hlthaff.2016.0005
7. Rosen DL, Grodensky CA, Holley TK. Federally-assisted healthcare coverage among male state prisoners with chronic health problems. PLoS One. 2016;11(8):e0160085. Medline:27479089 doi:10.1371/journal.pone.0160085
8. Knapp CD, Howell BA, Wang EA, Shlafer RJ, Hardeman RR, Winkelman TNA. Health insurance gains after implementation of the Affordable Care Act among individuals recently on probation: USA, 2008-2016. J Gen Intern Med. 2019;34(7):1086-1088. Medline:30815787 doi:10.1007/s11606-019-04900-3
9. Khatri UG, Howell BA, Winkelman TNA. Medicaid expansion increased medications for opioid use disorder among adults referred by criminal justice agencies. Health Aff (Millwood). 2021;40(4):562-570. Medline:33819101 doi:10.1377/hlthaff.2020.01251
10. Howell BA, Wang EA, Winkelman TNA. Mental health treatment among individuals involved in the criminal justice system after implementation of the Affordable Care Act. Psychiatr Serv. 2019;70(9):765-771. Medline:31138056 doi:10.1176/appi.ps.201800559
11. Howell BA, Hawks L, Wang EA, Winkelman TNA. Evaluation of changes in US health insurance coverage for individuals with criminal legal involvement in Medicaid expansion and nonexpansion states, 2010 to 2017. JAMA Health Forum. 2022;3(4):e220493. Medline:35977325 doi:10.1001/jamahealthforum.2022.0493
12. He Q, Barkowski S. The effect of health insurance on crime: evidence from the Affordable Care Act Medicaid expansion. Health Econ. 2020;29(3):261-277. Medline:31908077 doi:10.1002/hec.3977
13. Gutierrez CM, Pettit B. Employment and health among recently incarcerated men before and after the Affordable Care Act (2009-2017). Am J Public Health. 2020;110(S1):S123-S129. Medline:31967870 doi:10.2105/AJPH.2019.305419
14. Fry CE, McGuire TG, Frank RG. Medicaid expansion's spillover to the criminal justice system: evidence from six urban counties. RSF. 2020;6(2):244-263. Medline:33263082 doi:10.7758/rsf.2020.6.2.11

© 2024 Jolin JR et al. *JAMA Health Forum*.

15. Dickson MF, Staton M, Tillson M, Leukefeld C, Webster JM, Oser CB. The Affordable Care Act and changes in insurance coverage and source of health care among high-risk rural, substance-using, female offenders transitioning to the community. J Health Care Poor Underserved. 2018;29(3):843-863. Medline:30122668 doi:10.1353/hpu.2018.0064
16. Aslim EG, Mungan MC, Navarro CI, Yu H. The effect of public health insurance on criminal recidivism. J Policy Anal Manage. 2022;41(1):45-91. doi:10.1002/pam.22345
17. Howell BA, Hawks LC, Balasuriya L, Chang VW, Wang EA, Winkelman TNA. Health insurance and mental health treatment use among adults with criminal legal involvement after Medicaid expansion. Psychiatr Serv. 2023;74(10):1019-1026. Medline:37016823 doi:10.1176/appi.ps.20220171
18. Testa A, Porter LC. Previous incarceration, health insurance, and the Affordable Care Act in the U.S. Am J Prev Med. 2023;65(6):1034-1041. Medline:37380089 doi:10.1016/j.amepre.2023.06.014
19. Fone ZS, Friedson AI, Lipton BJ, Sabia JJ. Did the dependent coverage mandate reduce crime? J Law Econ. 2023;66(1):143-182. doi:10.1086/722461

© 2024 Jolin JR et al. *JAMA Health Forum*.
