# Supplementary material for: Access to Care and Outcomes With the Affordable Care Act for Persons With Criminal Legal Involvement: A Scoping Review
Source: JAMA Health Forum. 2024 Aug 23;5(8):e242640. doi: 10.1001/jamahealthforum.2024.2640 (PMC11344231; doi:10.1001/jamahealthforum.2024.2640)
Supplement: Supplement 2. — Data Sharing Statement [file jamahealthforum-e242640-s002.pdf]

Data Sharing Statement

Jolin. Access to Care and Outcomes With the Affordable Care Act for Persons With Criminal Legal Involvement. *JAMA Health Forum*. Published August 23, 2024.
doi:10.1001/jamahealthforum.2024.2640

Data
Data available: Yes
Data types: Data (not involving human participants)
How to access data: Data presented in Figure 2, Appendices, and References
When available: With publication

Supporting Documents
Document types: None

Additional Information
Who can access the data: The data will be available to everyone
Types of analyses: For any purpose
Mechanisms of data availability: Without investigator support
Any additional restrictions: None
